# Th17 Cell Response in SOD1^G93A^ Mice following Motor Nerve Injury

**DOI:** 10.1155/2016/6131234

**Published:** 2016-04-18

**Authors:** Allen Ni, Tao Yang, Nichole A. Mesnard-Hoaglin, Rafael Gutierrez, Evan B. Stubbs, Susan O. McGuire, Virginia M. Sanders, Kathryn J. Jones, Eileen M. Foecking, Junping Xin

**Affiliations:** ^1^Oncology Research Institute, Loyola University Chicago, Maywood, IL 60153, USA; ^2^Research Service, Department of Veterans Affairs, Edward Hines, Jr. VA Hospital, Hines, IL 60141, USA; ^3^Department of Brain Disease, Gansu Province Chinese Traditional Medicine Hospital, Lanzhou, Gansu 730050, China; ^4^Department of Ophthalmology, Stritch School of Medicine, Loyola University Chicago, Maywood, IL 60153, USA; ^5^Department of Molecular Virology, Immunology & Medical Genetics, College of Medicine, The Ohio State University, Columbus, OH 43210, USA; ^6^Department of Anatomy and Cell Biology, School of Medicine, Indiana University, Indianapolis, IN 46202, USA; ^7^Research and Development Service, Roudebush VA Hospital, Indianapolis, IN 46202, USA; ^8^Department of Otolaryngology, Loyola University Medical Center, Maywood, IL 60153, USA; ^9^Department of Molecular Pharmacology and Therapeutics, Loyola University Chicago, Maywood, IL 60153, USA

## Abstract

An increased risk of ALS has been reported for veterans, varsity athletes, and professional football players. The mechanism underlying the increased risk in these populations has not been identified; however, it has been proposed that motor nerve injury may trigger immune responses which, in turn, can accelerate the progression of ALS. Accumulating evidence indicates that abnormal immune reactions and inflammation are involved in the pathogenesis of ALS, but the specific immune cells involved have not been clearly defined. To understand how nerve injury and immune responses may contribute to ALS development, we investigated responses of CD4^+^ T cell after facial motor nerve axotomy (FNA) at a presymptomatic stage in a transgenic mouse model of ALS (B6SJL SOD1^G93A^). SOD1^G93A^ mice, compared with WT mice, displayed an increase in the basal activation state of CD4^+^ T cells and higher frequency of Th17 cells, which were further enhanced by FNA. In conclusion, SOD1^G93A^ mice exhibit abnormal CD4^+^ T cell activation with increased levels of Th17 cells prior to the onset of neurological symptoms. Motor nerve injury exacerbates Th17 cell responses and may contribute to the development of ALS, especially in those who carry genetic susceptibility to this disease.

## 1. Introduction

An increased risk of ALS is associated with certain populations who have a history of extensive physical contact such as varsity athletics, professional soccer players, and military veterans [1–3]. Motor nerve injury as a trigger to degeneration has been proposed in these populations, but the underlying mechanism remains elusive. In order to investigate this hypothesis, we utilized the motor nerve injury (facial nerve axotomy (FNA)) in the ALS mouse model (SOD1^G93A^ mice) to evaluate the impact of FNA on motoneuron survival after injury. We found that FNA-induced motor neuron loss is significantly increased in SOD1^G93A^ mice relative to WT mice. Importantly, the increased motor neuron loss in SOD1^G93A^ mice can be prevented by adoptive transfer of immune cells from wild-type mice [4]. These data suggest that individuals with a genetic susceptibility to ALS are more vulnerable to nerve injury-induced neurodegeneration. Because such vulnerability is impacted by the immune system, we hypothesize that FNA may induce a more pronounced proinflammatory response in SOD1^G93A^ mice than in WT mice, which in turn impairs the function of neuroprotective immune responses [4].

As the pivotal cell of immunoregulation, the CD4^+^ T cell has been of a great interest in the investigation of the pathogenesis of ALS. CD4^+^ T cells have several subsets with distinct immunoregulatory functions. In late-stage ALS patients, the total number of naïve CD4^+^ T cells is decreased and CD4^+^ T cell infiltration in the spinal cord and brain is significantly increased [5, 6]. In addition, elevated Th1 cells in cerebrospinal fluid and elevated IL-17 and Th17-related cytokines (IL-6, TNF-*α*, IL-1, and IL-23) [7–9] in the serum have also been observed in ALS patients. Furthermore, the level of antineuroinflammatory subsets, Th2 and Treg cells [10, 11], appear to regulate the speed of disease progression. However, the roles of proneuroinflammatory subsets, Th1 and Th17 cells, in promoting ALS development has yet to be established due to two challenges: late diagnosis and the chronic nature of the disease. The late diagnosis makes it difficult to conclude whether observed abnormal immune response and inflammation are the cause or the result of the disease. The chronic nature of ALS also makes it difficult to determine the best timing for the detection of such autoimmune responses.

We have previously demonstrated that FNA is capable of inducing a readily detectable immune response in a predictable time period (7–14 days) [12]. In the current study, we performed FNA in presymptomatic B6SJL SOD1^G93A^ mice (8-week-old) and examined CD4^+^ T responses in a time course after FNA. We found that abnormal CD4^+^ T cell activation with increased Th17 cells is present in SOD1^G93A^ mice prior to the onset of neurological symptoms. FNA further exacerbates CD4^+^ T cell activation and Th17 cell responses in SOD1^G93A^ mice. These results suggest that SOD1^G93A^ mice have impaired immunoregulatory mechanisms that normally dampen injury-induced inflammatory responses and that Th17 cell-promoted inflammation might contribute to the increase in injury-induced motoneuron death in SOD1^G93A^ mice.

## 2. Materials and Methods

### 2.1. Animals and Surgical Procedures

Six-week-old female B6SJL SOD1^G93A^ and wild-type female B6SJLF1/J mice were obtained from Jackson Laboratory (Sacramento, CA, USA). All mice were housed and surgery was performed as previously described [4, 12]. All surgical procedures were completed in accordance with National Institutes of Health guidelines on the care and use of laboratory animals for research purposes.

### 2.2. Preparation of CD4^+^ T Cells

The right cervical lymph nodes (draining cervical lymph node (dCLN)) were collected from uninjured (control) or axotomized mice (*n* = 4/group) at 7, 9, and 14 days postaxotomy (dpa). CD4^+^ T cells were isolated via autoMACS using anti-CD4 magnetic beads as previously described [4, 12].

### 2.3. Flow Cytometric Staining and Analysis

CD4^+^ T cells separated from the draining cervical lymph node preparation were incubated for 6 hours with phorbol myristate acetate (PMA, 50 ng/mL) and ionomycin (500 ng/mL, P/I, Sigma, St. Louis, MO) with brefeldin A (BFA, 10 *μ*g/mL, Sigma, St. Louis, MO) added during the final 2 hours. For the intracellular staining, CD4^+^ T cells were permeabilized with saponin (1 mg/mL, Sigma, St. Louis, MO) and double-stained with two of the following antigens: anti-CD4-eFluor 450, anti-IFN-*γ*-Alexa Fluor-488, anti-IL-17A-PE-Cy7, anti-TNF-*α*-PE, anti-IL-10-PerCp-Cy5.5 and anti-IL-4-APC, or anti-CD8-FITC. For the immune cells subsets identification, freshly collected dCLN cells were stained with anti-CD3-PE, anti-B220-APC-Cy7, anti-CD4-FITC, and anti-CD8-APC. For identification of activation, cells were stained with anti-CD4-eFluor 450, anti-CD69-PerCp-Cy5.5, anti-CD44-APC, and anti-CD62L-FITC. The stained cells were analyzed using a BD LSRFortessa*™* FACS machine with Flowjo software. All antibodies were purchased from eBioscience (San Diego, CA).

### 2.4. Statistical Analysis

Data are expressed as mean ± standard deviation (SD). A one-way ANOVA with the Bonferroni post hoc test was used for comparisons of two specific groups. Differences were considered significant at *p* < 0.05.

## 3. Results

### 3.1. Enhanced Immune Responses to Facial Nerve Injury in SOD1^G93A^ Mice

**Figure 1:**
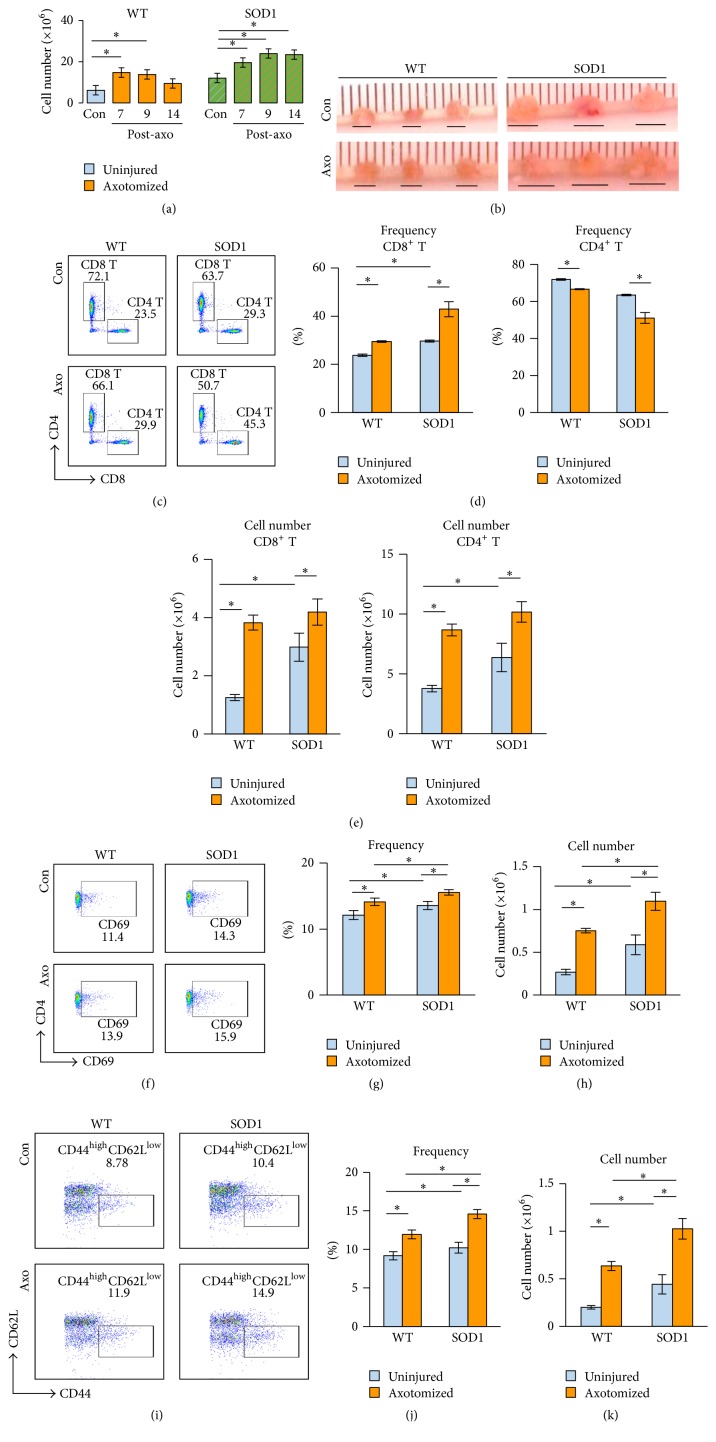
Inflammation and CD4 T cell activation in SOD1^G93A^ mice. (a) Cervical lymph nodes were collected from uninjured (Con) and axotomized WT and SOD1^G93A^ mice at 7, 9, and 14 dpa. The average of total number of dCLN from one mouse was calculated and compared (*n* = 4 mice/group). (b) dCLN from 3 individual mice in each group were collected and photographed on 7 dpa (the underline denotes the horizontal dimension of each dCLN). (c) dCLN were gated on T cells (CD3^+^ cells) and further analyzed for their subsets: CD4^+^ and CD8^+^ T cells. (d) the average of CD4^+^ and CD8^+^ T cell percentages in each group was calculated and compared. (e) The total numbers of CD4^+^ and CD8^+^ T cells were calculated on the basis of the total number of corresponding dCLN and their percentages. (f) dCLN cells were gated on CD4^+^ T cells and analyzed for CD69^+^ cells (in the square gate). (g) the average of CD69^+^ cell percentages in each group was calculated and compared. (h) The total number of CD69^+^ cells was calculated on the basis of the total number of corresponding dCLN and their percentages. (i) dCLN cells were gated on CD4^+^ T cells and analyzed for CD62L^low^CD44^high^ cells (in the square gate). (j) the average of CD62L^low^CD44^high^ cell percentages in each group was calculated and compared. (k) The total number of CD62L^low^CD44^high^ cells was calculated on the basis of the total number of corresponding dCLN and their percentages (data are presented as mean ± SD; *n* = 4 mice/group, ^*∗*^ *p* < 0.05; blue bars represent uninjured animals, whereas gold bars represent animals that have been subjected to axotomy).

Head injury is associated with an increased risk for developing ALS [1–3], leading us to hypothesize that inappropriate activation of the immune system from prior injury may underlie the development of ALS. Therefore, in the current study, we used the FNA model of motor neuron injury to compare immune responses in WT versus SOD1^G93A^ mice which serve as a mouse model of ALS to examine underlying alterations in immune activation and implications for disease development in SOD1^G93A^ mice (presymptomatic, 8-week-old B6SJL). As shown in Figure 1(a), basal numbers (prior to the FNA) of total cells recovered from one dCLN WT mouse were 6.13 ± 0.44 (×10^6^) versus 12.1 ± 0.99 (×10^6^) for SOD1^G93A^ mice, suggesting that SOD1^G93A^ mice have greater baseline number of lymphocytes than do WT mice. Following FNA, a transient increase in the number of total cells recovered was noted in WT mice and returned to basal levels at 14 days after FNA. In contrast, SOD1^G93A^ mice showed a progressive and sustained increase in total cell numbers in the dCLN. Differences in cell counts correlated with the size of dCLN in these mice (Figure 1(b)).

To further differentiate T cell subsets, we analyzed the percentage of CD4^+^ versus CD8^+^ T cells (Figures 1(c)–1(e)). Prior to the FNA, both WT and SOD1^G93A^ mice had a ratio of CD4 : CD8 that was approximately 2 : 1. Although the ratio of CD4 : CD8 remained close to 2 : 1 in WT mice following FNA, it decreased to a ratio approaching 1 : 1 in SOD1^G93A^ mice (Figures 1(c)-1(d)). However, because total cell numbers in the dCLN increased after FNA, the absolute number of both CD4^+^ and CD8^+^ increased in both WT and SOD1^G93A^ (Figure 1(e)). These data suggest that an enhanced basal level of inflammation in SOD1^G93A^ mice may impair immunoregulatory mechanisms that normally dampen injury-induced inflammatory responses before the onset of neurological symptoms.

### 3.2. Increased CD4^+^ T Cell Activation in Response to FNA

CD4^+^ T cells play a crucial role in regulation of the immune response. Accordingly, we examined further the activation status of CD4^+^ T cell responses at 7 days after FNA, the peak time of CD4^+^ T cell response [12]. In the WT mice, T cells at both the early activation stage (CD69^+^, Figures 1(f)–1(h)) and effector stage (CD62L^low^CD44^high^, Figures 1(i)–1(k)) were increased after FNA, as reflected by frequency (Figures 1(g) and 1(j)) and total number (Figures 1(h) and 1(k)). The same pattern was also found in SOD1^G93A^ mice, but at a higher magnitude. In addition, FNA-induced activation levels of CD4^+^ T cells in WT mice were comparable to that of SOD1^G93A^ mice prior to the FNA in terms of both percentage and total number, suggesting that the FNA-induced activation of T cells in WT mice may occur in a similar manner as T cell activation in SOD1^G93A^ mice, prior to disease onset.

### 3.3. Th17 Cell Response in SOD1^G93A^ Mice

**Figure 2:**
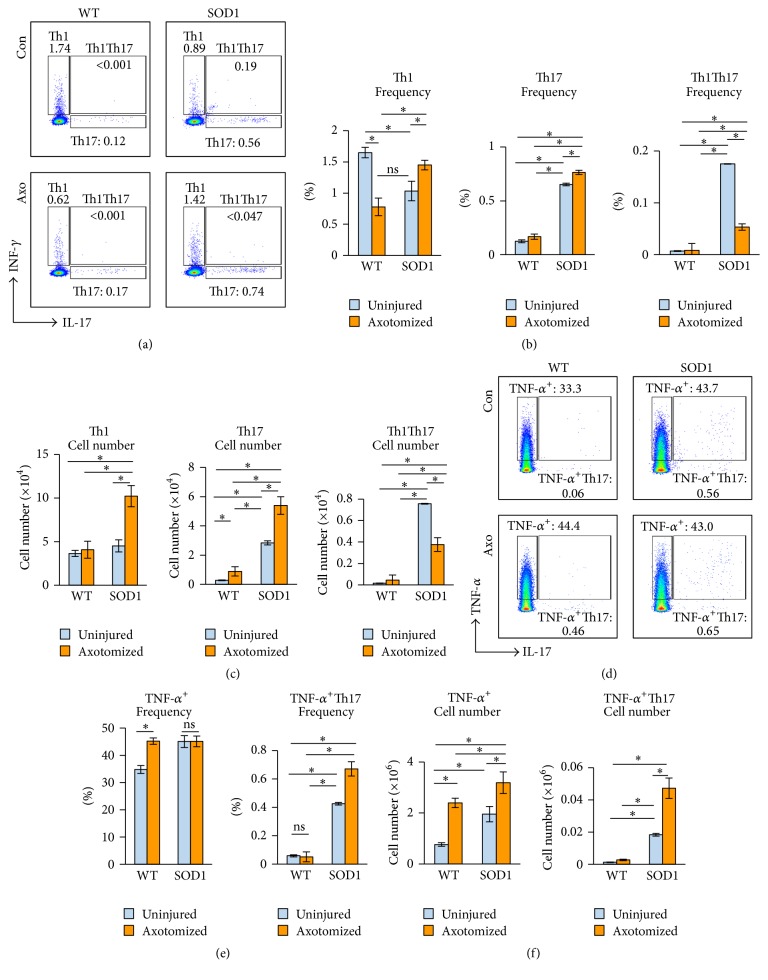
Th17 cell responses in SOD1^G93A^ mice. CD4^+^ T cells were isolated from dCLN from uninjured (Con) and axotomized WT and SOD1^G93A^ mice at 7 dpa (*n* = 4 mice/group). (a) Intracellular staining was performed for IFN-*γ* and IL-17 and analyzed using FACS. (b) The averaged percentages of Th1, Th17, and Th1Th17 cells in each groups were calculated and compared. (c) The averaged total number of Th1, Th17, and Th1Th17 cells in each groups was calculated on the basis of the total number of corresponding dCLN and their percentages. (d) Intracellular staining was performed for TNF-*α* and IL-17 and analyzed using FACS. (e) The averaged percentages of TNF-*α* ^+^ and TNF-*α* ^+^Th17 cells in each groups were calculated and compared. (f) The averaged total number of TNF-*α* ^+^ and TNF-*α* ^+^Th17 cells in each groups was calculated on the basis of the total number of corresponding dCLN and their percentages (data are presented as mean ± SD; *n* = 4 mice/group, ^*∗*^ *p* < 0.05; blue bars represent uninjured animals, whereas gold bars represent animals that have been subjected to axotomy).

CD4^+^ T subsets have distinct functions in terms of neuroprotective or neurodestructive effects [10–15]. Therefore, we examined dCLN CD4^+^ T subsets in WT and SOD1^G93A^ mice at 7 days after FNA by staining intracellularly for IFN-*γ* (Th1 cells), IL-17 (Th17 cells), TNF-*α* (pro-inflammatory cells), IL-4 (Th2 cells), and IL-10 (Treg cells). Following FNA, the frequency of Th1 cells in the WT mice was decreased, although the total number did not significantly change. In contrast, both the frequency and total number of Th1 cells were significantly increased in SOD1^G93A^ mice (Figures 2(a)-2(b),* first column*). In addition, Th17 cells in the WT mice did not change in frequency and increased only slightly in total number. In contrast, SOD1^G93A^ mice possessed both a greater frequency and more total Th17 cells than did WT mice, regardless of FNA state. In fact, the frequency of Th17 cells was 3-fold higher (0.65 ± 0.05% versus 0.13 ± 0.03%) prior to injury and was 4-fold higher (0.77 ± 0.02% versus 0.16 ± 0.02%) after FNA (Figure 2(b),* middle column*) in SOD1^G93A^ mice, whereas the numbers of total Th17 cells were 9-fold higher [2.8 ± 0.31 versus 0.28 ± 0.03 (×10^4^)] prior to injury and 5-fold higher [5.39 ± 0.61 versus 0.89 ± 0.14 (×10^4^)] after FNA (Figure 2(c) *middle column*) in SOD1^G93A^ mice. In the WT mice, the frequency and total number of Th1Th17 cells were similar prior to and after FNA. In contrast, the frequency and total number of Th1Th17 cells in SOD1^G93A^ mice decreased after FNA. We did not find significant difference of Th2 and Treg cells between WT and SOD1^G93A^ mice prior to or after FNA (*data not shown*).

Further analysis of TNF-*α* expression in CD4^+^ T cells revealed that both frequency and percentage of TNF-*α*-single-positive cells were significantly increased in the WT mice following FNA. However, in SOD1^G93A^ mice, though the percentage did not change after FNA, the total number of TNF-*α* ^+^ cells significantly increased (Figures 2(e) and 2(f),* left column*). TNF-*α*-expressing Th17 (TNF-*α* ^+^Th17) in WT mice was low in both frequency and total number and did not significantly change after FNA. In contrast, TNF-*α* ^+^Th17 in SOD1^G93A^ mice had a higher basal frequency and total number which was further increased in response to FNA (Figures 2(e) and 2(f),* right column*). These data suggest that TNF-*α*-expressing Th17 cells might be an important subset of autoimmune cells involved in injury-induced inflammatory damage in SOD1^G93A^ mice.

## 4. Discussion

We have previously demonstrated that CD4^+^ T cells mediate neuroprotection after nerve injury [4, 16]. In ALS, CD4^+^ T cells also play an important role in restricting disease progression [13, 14]; however, this neuroprotection is in a context- and subset-dependent manner [10–15]. For example, anti-inflammatory subsets of CD4^+^ T cells are generally thought to be the types of neuroprotective immune cells which support facial motoneuron survival after nerve injury and may therefore slow down the disease progression in ALS [10, 11]. Our previous studies revealed that multiple subsets of CD4^+^ T cells develop following FNA in WT mice, including both anti-inflammatory and proinflammatory subsets of CD4^+^ T cells [12]. We hypothesize that the balance between these subsets of CD4^+^ T is critical for the resolution of necessary and beneficial inflammation as well as induction of repair tissue and support mechanisms for survival of damaged motoneurons. In the current work, we used the SOD1^G93A^ mice, an ALS mouse model, to show that FNA induces significant motoneuron loss relative to WT mice and that this loss is similar to that of immunodeficient mice (Rag2^−/−^ mice) [4, 16]. Exacerbation of FNA-induced motoneuron loss in SOD1^G93A^ mice may result from a poorly controlled inflammatory response to injury, increased basal levels of inflammation inherent to this model or perhaps to failure of development of neuroprotective CD4^+^ T subsets [4] as the current study revealed that there are significant differences of FNA-induced immune responses between WT and SOD1^G93A^ mice.

First, we found that while WT mice mounted a well-controlled immune response in the draining lymph nodes following FNA, SOD1^G93A^ mice had an enhanced and persistent immune response as indicated by the enlarged size of the draining lymph nodes as well as an increase in total cell numbers. Further analysis for T cell subset responses revealed that the ratio of CD4 : CD8 T cells in WT mice did not change in response to FNA, whereas the ratio decreased in SOD1^G93A^ mice. Because CD4^+^ T cells regulate CD8 T cell responses and CD4^+^ T cells are the major type of T cells that impact the neuronal survival after FNA, we focused on CD4^+^ T cell responses in the analysis of T cell activation and subsets. Our data indicate that activation of CD4^+^ T cells is higher in SOD1^G93A^ than WT mice. Regarding the subsets, we did not find significant differences in anti-inflammatory T cell subsets (Th2 and Treg cells) between WT and SOD1^G93A^ mice either prior to or after FNA; however, significant differences were noted in the proinflammatory subsets (Th1 and Th17 cells) between these two types of mice. Without nerve injury, Th1 cell frequency and total number were similar in WT and SOD1^G93A^ mice; however, FNA induced an increase in Th1 total number in SOD1^G93A^ mice but not in WT mice. In the uninjured state, both Th17 cell frequency and total number were higher in SOD1^G93A^ mice than in WT mice. Further increases in Th17 cell frequency and total number were noted in SOD1^G93A^ mice but not in WT mice under FNA injury conditions. Importantly, Th17 cells are also TNF-*α*-expressing cells in SOD1^G93A^ mice but not in WT mice. These data collectively indicate that feed-forward proinflammatory response to injury occurs in SOD1^G93A^ mice.

We recently demonstrated that whole splenocytes, but not isolated CD4^+^ T cells, from WT mice can reduce FNA-induced facial motor nucleus (FMN) loss in SOD1^G93A^ mice [4]. In addition, isolated CD4^+^ T cells, but not whole splenocytes collected from SOD1^G93A^ mice, are capable of supporting FMN survival in immunodeficient mice (Rag2^−/−^) [4]. These data suggest that the microenvironment in SOD1^G93A^ mice may direct CD4^+^ T cell differentiation into a neurodestructive subset. Consistent with this hypothesis, the data from the current study suggest that FNA-induced Th17 cell responses in SOD1^G93A^ mice may exacerbate neuroinflammation, without a concomitant induction of the neural repair phase, which in turn results in increased motoneuron death.

## 5. Conclusion

Enhanced CD4^+^ T cell activation and Th17 cell responses in SOD1^G93A^ mice exist prior to the onset of overt neurological symptoms. Motor nerve injury further increases CD4^+^ T cell activation and Th17 cell responses in SOD1^G93A^ mice. We therefore propose that cell-promoted inflammation may be involved in the motoneuron death during ALS disease onset and progression.

## Abbreviations

FMN: Facial motoneuron
WT: Wild-type
SOD1^G93A^: Copper zinc superoxide dismutase 1
dpa: Days post axotomy
dCLN: Cervical lymph node
Th: T helper cell
TNF: Tumor necrosis factor
FNA: Facial nerve axotomy.
